# The loss of *LRPPRC* function induces the mitochondrial unfolded protein response

**DOI:** 10.18632/aging.100812

**Published:** 2015-09-26

**Authors:** Fabian Köhler, Anne Kathrin Müller-Rischart, Barbara Conradt, Stéphane Guy Rolland

**Affiliations:** ^1^ Center for Integrated Protein Science, Fakultät für Biologie, Ludwig-Maximilians-Universität München, 82152 Planegg-Martinsried, Germany

**Keywords:** Mitochondrial stress response, UPR^mt^, mitochondrial hyperfusion, mma-1, LRPPRC

## Abstract

The inactivation of the *LRPPRC* gene, which has previously been associated with the neurodegenerative French Canadian Leigh Syndrome, results in a decrease in the production of mitochondria-encoded subunits of complex IV, thereby causing a reduction in complex IV activity. Previously we have shown that reducing complex IV activity triggers a compensatory and conserved mitochondrial hyperfusion response. We now demonstrate that *LRPPRC* knock-down in mammalian cells leads to an imbalance between mitochondria-encoded and nuclear-encoded subunits of complex IV and that this imbalance triggers the mitochondrial unfolded protein response (UPR^mt^). The inactivation of the *LRPPRC*-like gene *mma-1* in *C. elegans* also induces UPR^mt^, which demonstrates that this response is conserved. Furthermore, we provide evidence that mitochondrial hyperfusion and UPR^mt^ are coordinated but mediated by genetically distinct pathways. We propose that in the context of *LRPPRC mma-1* knock-down, mitochondrial hyperfusion helps to transiently maintain mitochondrial ATP production while UPR^mt^ participates in the restoration of mitochondrial proteostasis. Mitochondrial proteostasis is not only critical in pathophysiology but also during aging, as proteotoxic stress has been shown to increase with age. Therefore, we speculate that the coordination of these two mitochondrial stress responses plays a more global role in mitochondrial proteostasis.

## INTRODUCTION

Mitochondria are essential eukaryotic organelles that participate in processes such as cellular energy production, cell signaling and apoptosis [1-3]. The production of cellular energy by mitochondria produces as a byproduct reactive oxygen species (ROS). While the effect of ROS on the accumulation of mutations in the mitochondrial genome is still under debate, ROS have been shown to promote protein oxidation and consequently misfolding and/or unfolding of these proteins inside mitochondria [4, 5]. The resulting proteotoxic stress has been shown to increase with age and to participate in several age-related disorders, such as neurodegenerative diseases [6]. To maintain mitochondrial proteostasis and, hence, mitochondrial function, it is important that damaged proteins are eliminated by mitochondrial proteases and that mitochondrial chaperones assist in the folding of nascent proteins. Another challenge that mitochondria face is the fact that the assembly of complexes of the Electron Transport Chain (ETC) in the inner mitochondrial membrane (IMM) requires a proper stoichiometric ratio of their subunits. Hence, misfolding of one subunit of an ETC complex results in the failure to assemble the entire complex. Consequently, the other subunits will accumulate in the mitochondrial matrix and thereby compromise mitochondrial proteostasis. Finally, ETC components are encoded by the mitochondrial and the nuclear genome. In order to maintain their respective stoichiometric ratios, the expression of mitochondrial and nuclear encoded proteins therefore has to be properly coordinated [7].

Several mitochondrial stress response pathways that maintain mitochondrial function have been described. One or more of these pathways are activated, depending on the extent to which mitochondrial proteostasis is compromised or mitochondria are damaged. For example, severely damaged mitochondria, which have irremediably lost their membrane potential, are eliminated from the functional mitochondrial network by mitochondrial-specific autophagy (mitophagy) (see for review [8]). The accumulation of unfolded or misfolded mitochondrial proteins activates the mitochondrial unfolded protein response (UPR^mt^). This response leads to the increased production and import into mitochondria of chaperones (such as HSP60 and HSP70) and proteases (such as ClpP), which help misfolded proteins to fold properly or cause their degradation, respectively (see for review [9]). Finally, the mitochondrial hyperfusion response has recently been described in mammalian cells and in *C. elegans.* Various forms of stress, such as a decrease in complex IV activity, the inhibition of cytosolic protein synthesis or starvation, induce mitochondrial hyperfusion in order to maintain mitochondrial ATP production [10-12].

The human *LRPPRC* gene encodes a leucine-rich pentatricopeptide repeat containing protein that is imported into mitochondria and that is mutated in patients with French Canadian Leigh Syndrome, a neurodegenerative disorder associated with complex IV deficiency [13]. Inside the mitochondrial matrix, the LRPPRC protein is part of a ribonucleoprotein complex that post-transcriptionally controls the expression of specific mitochondrial mRNAs such as the mRNA coding for COX I, a component of complex IV [14, 15]. We have previously shown that reducing *LRPPRC* function in mammalian tissue culture cells or reducing the function of the *LRPPRC*-like gene *mma-1* (*mma*, mitochondrial morphology-abnormal) in *C. elegans* leads to a decrease in the level of COX I and, consequently, a decrease in complex IV activity [11]. This decrease in complex IV activity is compensated by an evolutionarily conserved mitochondrial hyperfusion response [11]. COX I is one of three mitochondria-encoded subunits of complex IV. The remaining 11 subunits in mammals and 6 subunits in *C. elegans* are nuclear-encoded [16, 17]. Therefore, we hypothesized that the reduction of COX I protein level upon *LRPPRC mma-1* knock-down might cause an imbalance between nuclear- and mitochondria-encoded subunits and, hence, trigger UPR^mt^. Here we report that in addition to triggering a mitochondrial hyperfusion response, *LRPPRC mma-1* knock-down also triggers UPR^mt^ in both mammalian cell cultures and *C. elegans*. We propose that these two responses act together to maintain and restore mitochondrial function, in response to decreased complex IV activity.

## RESULTS

### *LRPPRC* siRNA leads to an imbalance between mitochondria-encoded and nuclear-encoded sub-units of complex IV and triggers UPR^mt^

**Figure 1:**
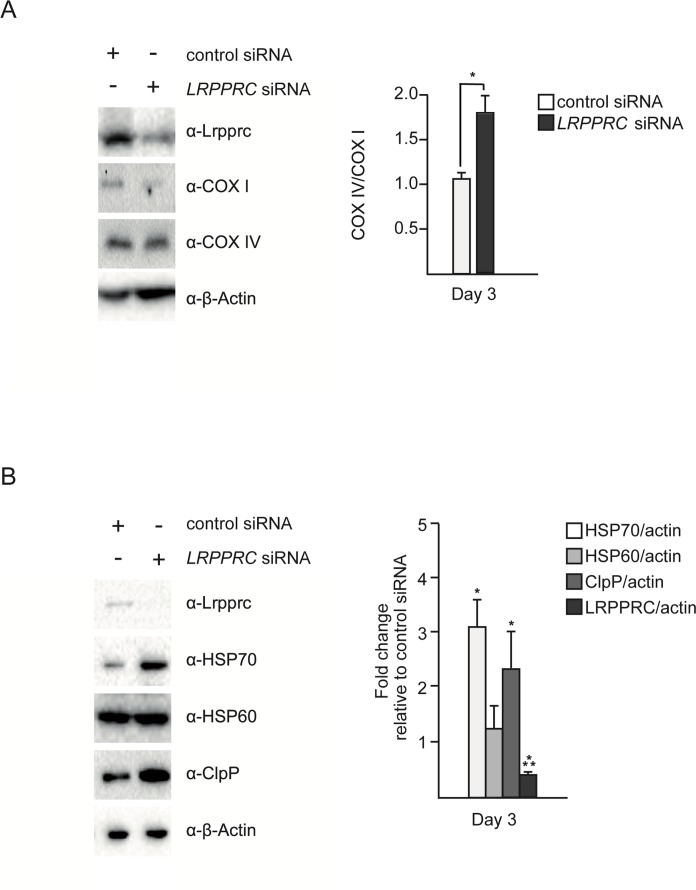
Silencing of *LRPPRC* leads to an imbalance between mitochondria-encoded and nuclear-encoded subunits of complex IV and triggers UPR^mt^ SH-SY5Y cells were treated with control or *LRPPRC* siRNA for three days and transferred for 24 hours to low glucose medium (5 mM) to enhance oxidative phosphorylation. (**A**) Total protein extracts were analyzed by Western using anti-LRPPRC, anti-COX I, anti-COX IV and anti-β-Actin antibodies. COX IV/COX I ratios are indicated. (**B**) The same protein extracts as in panel **A** were also analyzed by Western using anti-LRPPRC, anti-HSP70, anti-HSP60, anti-ClpP and anti-β-Actin antibodies. Ratios relative to the control siRNA are indicated. (For all panels, quantifications are based on data from three independent experiments; average values are shown and error bars indicate s.d.; *p ≤ 0.05, ***p ≤ 0.001 by one way ANOVA for panel **A** and one sample *t*-test for panel **B**).

Inactivation of *LRPPRC* results in a decrease in the production of mitochondria-encoded subunits of complex IV [11, 14, 15]. We reasoned that this decrease may lead to an imbalance between mitochondria-encoded and nuclear-encoded subunits of this complex and thereby trigger UPR^mt^. To test this hypothesis, we inactivated *LRPPRC* in SH-SY5Y cells using small interfering RNA (siRNA) and quantified the level of COX I protein (a mitochondria-encoded subunit of complex IV) and the level of COX IV protein (a nuclear-encoded subunit of complex IV). As shown in Figure 1A, the level of COX I protein decreases after 3 days of *LRPPRC* siRNA (down to 50% of the level in control siRNA cells). In contrast, the level of COX IV protein remains stable, resulting in an imbalance between COX I and COX IV subunits with ~1.8 times more COX IV subunits than COX I subunits upon *LRPPRC* siRNA.

In order to test whether this imbalance triggers UPR^mt^, we measured the levels of the mitochondrial chaperones HSP60 and HSP70 as well as the mitochondrial protease ClpP. We observed that 3 days of *LRPPRC* siRNA triggers a ~3-fold increase in the level of endogenous HSP70 protein (Figure 1B; *n* = 3; *p* = 0.0473 by one sample *t*-test), a ~1.3-fold increase in the level of endogenous HSP60 protein (Figure 1B; *n* = 3; *p* = 0.178 by one sample *t*-test) and a ~2.3-fold increase in the level of the endogenous mitochondrial protease ClpP (Figure 1B; *n* = 3; *p* = 0.0202 by one sample *t*-test). Similar results were observed using HEK293T cells (Figure S1). Based on these results, we conclude that the imbalance between nuclear- and mitochondria-encoded subunits of complex IV caused by *LRPPRC* siRNA triggers UPR^mt^.

### The transient activation of UPR^mt^ by *LRPPRC* siRNA correlates with the restoration of the balance between complex IV subunits

**Figure 2:**
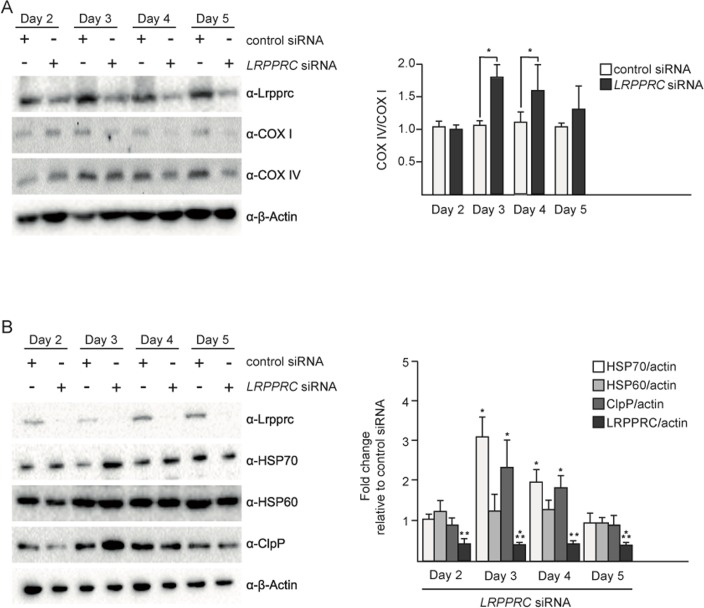
Upon *LRPPRC* siRNA-induced UPR^mt^, mitochondrial proteostasis is restored UPR^mt^ in SH-SY5Y cells treated with control or *LRPPRC* siRNA for 2, 3, 4 or 5 days and transferred for 24 hours in low glucose medium. (**A**) Total protein extracts were analyzed by Western using anti-LRPPRC, anti-COX I, anti-COX IV and anti-β-Actin antibodies. COX IV/COX I ratios are indicated. (**B**) The same protein extracts as in panel A were also analyzed by Western using anti-LRPPRC, anti-HSP70, anti-HSP60, anti-ClpP and anti-β-Actin antibodies. Ratios relative to the control siRNA are indicated. (For all panels, quantifications are based on data from three independent experiments; average values are shown and error bars indicate s.d.; *p ≤ 0.05, **p ≤ 0.01, ***p ≤ 0.001 by one way ANOVA for panel **A** and one sample *t*-test for panel **B**).

In order to test the effect of the induction of UPR^mt^ upon *LRPPRC* siRNA on the balance between nuclear- and mitochondria-encoded complex IV subunits, we performed a time course experiment. The imbalance between COX I and COX IV is first observed after 3 days of *LRPPRC* siRNA (Figure 2A). This time point also corresponds to the highest level of induction of HSP70 and ClpP (Figure 2B). After 5 days of *LRPPRC* siRNA, the level of COX IV protein decreases (down to 50% of the level in control siRNA cells), restoring the balance between COX I and COX IV subunits (Figure 2A). This time point coincides with a decrease of the levels of HSP70 and ClpP back to the levels measured in control cells (Figure 2B). A similar transient activation of UPR^mt^ was observed in HEK293T cells (Figure S1). Therefore, the transient activation of UPR^mt^ correlates with the restoration of mitochondrial proteostasis in the context of *LRPPRC* siRNA.

### UPR^mt^ and mitochondrial hyperfusion are transient responses to *LRPPRC* siRNA that follow similar kinetics

We have previously shown that mitochondrial hyper-fusion is a transient response, which peaks at 3 days of *LRPPRC* siRNA (Figure S2; day 3: 50% of cells have hyperfused mitochondria, 32% have tubular mitochondria and 18% have fragmented mitochondria). After 4 or 5 days of inactivation, the population of cells that display hyperfused mitochondria decreases while the population of cells that display fragmented mitochondria increases (Figure S2; day 5: 35% of cells have hyper-fused mitochondria, 45% have tubular mitochondria and 28% have fragmented mitochondria). Similarly, we observed that the induction of UPR^mt^ is at its maximum at 3 days of *LRPPRC* siRNA, in both SH-SY5Y cells and HEK293T cells (Figure 2B, Figure S1). After 4 and 5 days of inactivation, the UPR^mt^ decreases in both cell lines (Figure 2B, Figure S1B). Hence, in mammalian cell culture, UPR^mt^ and the mitochondrial hyperfusion response both are transient and follow similar kinetics.

### Inactivation of the *LRPPRC*-like gene *mma-1* in *C. elegans* also induces UPR^mt^

**Figure 3:**
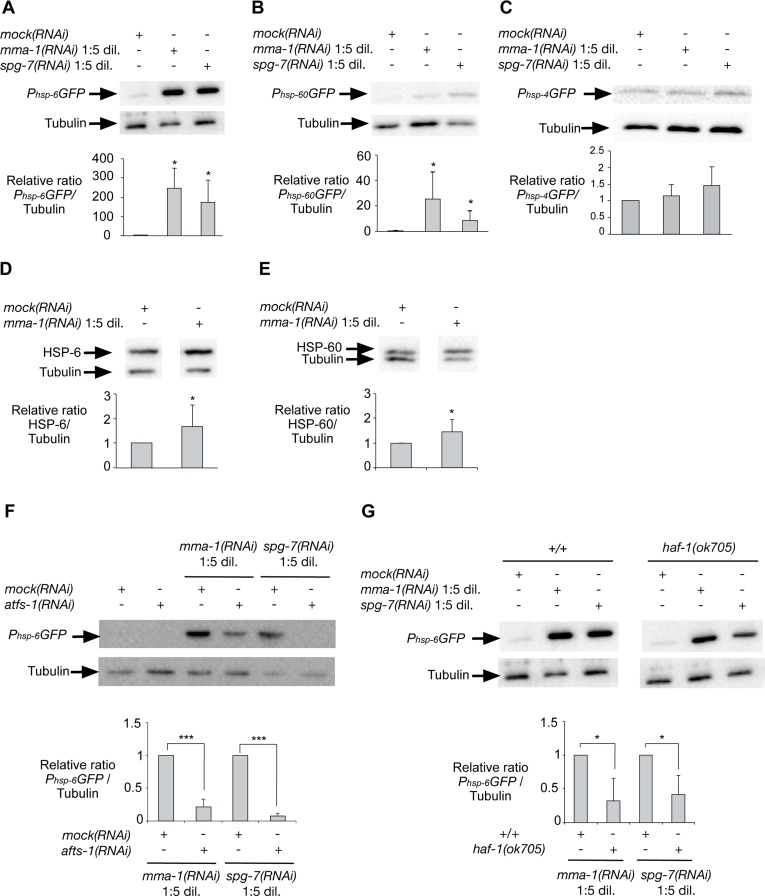
Inactivation of *mma-1* by RNAi in *C. elegans* induces ATFS-1-dependent UPR^mt^ Western analysis of (**A**) *P_hsp-6_GFP* (mitochondrial Hsp70), (**B**) *P_hsp-60_GFP* (mitochondrial Hsp60) or (**C**) *P_hsp-4_GFP* (ER BiP) reporter strains treated with *mock(RNAi)*, *mma-1(RNAi)* 1:5 dil. (diluted 1:5 (v/v) with *mock(RNAi)*) or *spg-7(RNAi)* 1:5 dil. (diluted 1:5 (v/v) with *mock(RNAi)*). Ratios of *P_hsp-6_GFP*/Tubulin, *P_hsp-60_GFP*/Tubulin and *P_hsp-4_GFP*/Tubulin relative to the *mock(RNAi)* treated animals are indicated (*n* = 5 for *P_hsp-6_GFP* + *mma-1(RNAi)*; *n* = 7 for *P_hsp-6_GFP* + *spg-7(RNAi)*; *n* = 6 for *P_hsp-60_GFP* + *mma-1(RNAi)*; *n* = 9 for *P_hsp-60_GFP* + *spg-1(RNAi)*; *n* = 5 for *P_hsp-4_GFP*). Western analysis of the effect of *mma-1(RNAi)* on endogenous (**D**) HSP-6 or (**E**) HSP-60 protein level. Ratios of HSP-6/Tubulin and HSP-60/Tubulin relative to the *mock(RNAi)* treated animals are indicated (*n* = 8 for HSP-6 and *n* = 10 for HSP-60). (**F**) Western analysis of the effect of ATFS-1 on *mma-1(RNAi)*-induced UPR^mt^. *mma-1(RNAi)* or *spg-7(RNAi)* were diluted either with *mock(RNAi)* or *atfs-1(RNAi)*. Relative ratios of *P_hsp-6_GFP*/Tubulin are indicated (*n* = 7 for *mma-1(RNAi)* and *n* = 10 for *spg-7(RNAi)*). (**G**) Western analysis of the effect of HAF-1 on *mma-1(RNAi)*-induced UPR^mt^. Wild-type *P_hsp-6_GFP* reporter strain (+/+) or *P_hsp-6_GFP* reporter strain carrying the *haf-1*(*ok705*) loss-of-function mutation were analyzed. The relative ratios of *P_hsp-6_GFP*/Tubulin are indicated (*n* = 5 for *mma-1(RNAi)* and *n* = 6 for *spg-7(RNAi)*) (For all panels, average values are shown and error bars indicate s.d.; *p ≤ 0.05, **p ≤ 0.01, ***p ≤ 0.001 by one sample *t*-test).

In order to address whether the induction of UPR^mt^ in response to *LRPPRC* siRNA is conserved, we inactivated the *LRPPRC*-like gene *mma-1* in *C. elegans*. We used the transcriptional reporters *P_hsp-6_GFP*, *P_hsp-60_GFP* and *P_hsp-4_GFP* to monitor the expression of the genes encoding the mitochondrial HSP70 chaperone (*hsp-6*), the mitochondrial HSP60 chaperone (*hsp-60*) or the endoplasmic reticulum (ER) chaperone BiP (*hsp-4*), respectively [18]. We reduced *mma-1* function using RNA-mediated interference (RNAi) by feeding [19]. Specifically, we grew L4 larvae of the reporter strains on RNAi plates seeded with an *Escherichia coli* strain expressing double-strand RNA (dsRNA) of *mma-1*. The plates were seeded with *mma-1(RNAi)* bacteria diluted 1:5 (v/v) with *mock(RNAi)* bacteria; hereafter referred to as ‘*mma-1(RNAi)* 1:5 dil.′. The expression of the reporters was analyzed four days later in the F1 generation. Using these conditions, the level of MMA-1 protein is reduced by ~40% (see below, Figure S4). As shown in Figure 3, the *P_hsp-6_GFP* reporter is strongly up-regulated in response to *mma-1(RNAi)* (on average ~200-fold compared to *mock(RNAi)*; Figure 3A; *n* = 5; *p* = 0.037 by one sample *t*-test). *P_hsp-60_GFP* expression was also increased in *mma-1(RNAi)* animals compared to *mock(RNAi)* animals, albeit to a lesser extent (Figure 3B; *n* = 9; *p* = 0.011 by one sample *t*-test). Finally, *mma-1* knock-down did not affect the expression of the ER chaperone *hsp-4* (Figure 3C; *n* = 5; *p* = 0.36 by one sample *t*-test). We also tested the effect of reducing the activity of the *C. elegans* homolog of paraplegin *spg-7*, which has previously been shown to induce UPR^mt^ [18]. Using the RNAi conditions described above, the inactivation of *spg-7* induces a strong up-regulation of the *P_hsp-6_GFP* reporter and a weak up-regulation of the *P_hsp-60_GFP* reporter but has no effect on the expression of the *P_hsp-4_GFP* reporter (Figure 3).

In order to confirm the effect of *mma-1(RNAi)* on the expression of *hsp-6* and *hsp-60* at the levels of endogenous HSP-6 and HSP-60 protein, we used a mouse monoclonal anti-HSP-60 antibody [20] and generated rabbit polyclonal anti-HSP-6 antibodies. We confirmed that the anti-HSP-6 antibodies specifically recognize HSP-6 (Figure S3). Using these antibodies we found that the inactivation of *mma-1* by RNAi leads to a ~1.7-fold increase in the level of endogenous HSP-6 protein (Figure 3D; *n* = 8; *p* = 0.024 by one sample *t*-test). In addition, we found that *mma-1(RNAi)* induces a ~1.4-fold increase in the level of endogenous HSP-60 protein (Figure 3E; *n* = 10; *p* = 0.022 by one sample *t*-test). The discrepancy between the level of induction for endogenous proteins (~1.4-fold and ~1.7-fold for HSP-60 and HSP-6, respectively) and for transcriptional reporters (~20-fold and ~200-fold for *P_hsp-60_GFP* and *P_hsp-6_GFP*, respectively) is most likely due to the copy number of the *P_hsp-6_GFP* and *P_hsp-60_GFP* transgenes integrated in the genome of the reporter strains; however, we cannot exclude that this discrepancy is also caused by post-transcriptional regulations of *hsp-6* and/or *hsp-60* expression. Based on these results we conclude that *mma-1(RNAi)* induces UPR^mt^ and the transcriptional up-regulation of the *hsp-6* and *hsp-60* genes, which results in a significant increase in the level of endogenous HSP-6 and HSP-60 protein.

### *mma-1(RNAi)*-induced up-regulation of *hsp-6* transcription is dependent on ATFS-1 and HAF-1

In *C. elegans*, UPR^mt^ is dependent on the peptide exporter HAF-1 as well as the bZip transcription factor ATFS-1 [21, 22]. Consistent with previous studies [22], we confirmed that the inactivation of *atfs-1* by RNAi suppresses the up-regulation of the *P_hsp-6_GFP* reporter induced by *spg-7(RNAi)* (Figure 3F; *n* = 7; *p* = 0.0001 by one sample *t*-test). Furthermore, we found that *mma-1(RNAi)*-induced UPR^mt^ is suppressed by *atfs-1(RNAi)* as well (Figure 3F; *n* = 10; *p* = 0.0001 by one sample *t*-test). To rule out that the effects observed are due to differences in RNAi efficiency, we quantified the knock-down of *mma-1* using anti-MMA-1 antibodies [11]. As shown in Figure S4A, the efficiency of *mma-1(RNAi)* in *atfs-1(RNAi)* animals is similar to that in wild-type animals (reduction by ~40% on average). Hence, the *mma-1(RNAi)*-induced up-regulation of *hsp-6* transcription is dependent on ATFS-1. In contrast, the *haf-1* loss-of-function mutation *ok705* only partially suppresses the up-regulation of *P_hsp-6_GFP* in response to *mma-1(RNAi)* (Figure 3G; *n* = 5; *p* = 0.0217 by one sample *t*-test). We observed a similar result for *spg-7(RNAi)*-induced *P_hsp-6_GFP* up-regulation (Figure 3G; *n* = 6; *p* = 0.017 by one sample *t*-test). We confirmed that the effects observed were not due to differences in the efficiency of *mma-1(RNAi)* (Figure S4B). Therefore, *mma-1(RNAi)* induces UPR^mt^ in a manner that is dependent on the bZip transcription factor ATFS-1 and that is partially dependent on the peptide exporter HAF-1.

### Strong depletion of MMA-1 protein leads to both mitochondrial hyperfusion and UPR^mt^ induction whereas mild depletion of MMA-1 protein only leads to UPR^mt^

**Figure 4:**
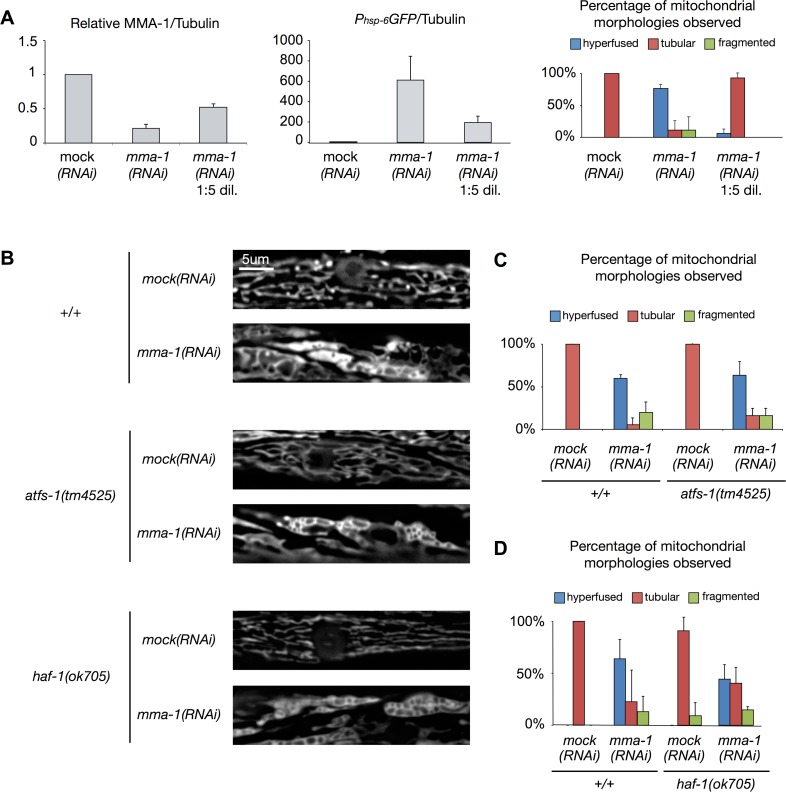
*mma-1(RNAi)*-induced mitochondrial hyperfusion is not dependent on ATFS-1 or HAF-1 (**A**) Strong depletion of MMA-1 protein (*mma-1(RNAi)*) leads to both mitochondrial hyperfusion and UPR^mt^ response whereas mild depletion of MMA-1 protein (*mma-1(RNAi)* 1:5 dil.) only leads to UPR^mt^. Average relative ratios of MMA-1/Tubulin and *P_hsp-6_GFP*/Tubulin are indicated. Mitochondrial morphology in body wall muscles was assessed in L4 larvae of the F1 generation. Percentage of animals showing fragmented, tubular or hyperfused mitochondria are indicated (*n* >10 for each condition; average values are shown and error bars indicate s.d.). (**B**) Fluorescence microscopy analysis of wild-type *P_myo-3_mitoGFP* reporter strain (+/+) or *P_myo-3_mitoGFP* reporter strain carrying the *haf-1(ok705)* mutation or *atfs-1(tm4525)* mutation. (**C-D**) Quantification of the different mitochondrial morphologies observed (at least 15 animals were analyzed for each condition; two independent experiments were performed for panel **C**; three independent experiments were performed for panel **D**; average values are shown and error bars indicate s.d.).

When growing animals on ‘non-diluted’ *mma-1(RNAi)* plates, we achieved on average an ~80% reduction in the level of MMA-1 protein (Figure 4A; *mma-1(RNAi)*). Under these conditions, as previously reported, animals exhibit hyperfused mitochondria in body wall muscle cells (76%; Figure 4A; *n* = 17; two independent experiments) [11]. These animals also show a stronger UPR^mt^ as reflected by the up-regulation of the *P_hsp-6_GFP* reporter (on average ~600-fold compared to *mock(RNAi)* animals; Figure 4A). When reducing the level of MMA-1 protein by only 40% (*mma-1(RNAi)* 1:5 dil.), the *P_hsp-6_GFP* reporter is still up-regulated (on average 200-fold compared to *mock(RNAi)* animals; Figure 4A); however, these animals do not exhibit mitochondrial hyperfusion (6%; Figure 4A; *n* = 16; two independent experiments). Hence, mild depletion of MMA-1 protein leads to the activation of the UPR^mt^ pathway and strong depletion of MMA-1 protein additionally leads to the activation of the mitochondrial hyperfusion response pathway.

### *mma-1(RNAi)*-induced mitochondrial hyperfusion is independent of HAF-1 and ATFS-1

In order to test whether the mitochondrial hyperfusion response induced by the inactivation of *mma-1* is dependent on HAF-1 and ATFS-1, we used a transgene (*bcIs78* [*P_myo-3_mitoGFP*]) that expresses mitochondrial matrix-targeted GFP in body wall muscles, which allows us to monitor steady-state mitochondrial morphology in these cells [11]. In an otherwise wild-type background, *mma-1(RNAi)* causes mitochondrial hyperfusion (64%, *n* = 30, Figure 4B-D, three independent experiments) [11]. Similarly, *mma-1(RNAi)* causes mitochondrial hyperfusion in animals homozygous for the *atfs-1* loss-of-function mutation *tm4525* (67%, Figure 4B-C; *n* = 19, two independent experiments). Finally, *mma-1(RNAi)* also causes mitochondrial hyperfusion in animals homozygous for the *haf-1* loss-of-function mutation *ok705* (45%; Figure 4B-D; *n* = 33; three independent experiments). Compared to the response in the wild-type and *atfs-1*(*tm4525*) background, the response in the *haf-1*(*ok705*) background is slightly reduced; however, the difference is not statistically significant (*p* = 0.22 by Student *t*-test). Based on these results we conclude that *mma-1(RNAi)*-induced mitochondrial hyperfusion is independent of ATFS-1 and HAF-1.

### ATFS-1-dependent UPR^mt^ is not essential for viability in response to *mma-1(RNAi)*

We previously showed that *mma-1(RNAi)* causes synthetic embryonic lethality and synthetic midlarval arrest in the *fzo-1(tm1133)* loss-of-function mutant background, indicating that mitochondrial hyperfusion is essential for viability in response to *mma-1(RNAi)* [11]. To test whether UPR^mt^ is also essential for viability in response to *mma-1(RNAi)*, we inactivated *mma-1* in animals carrying a loss-of-function mutation in the *atfs-1* gene (*atfs-1(tm4525)*) [22]. We found that *mma-1(RNAi)* does not cause any obvious embryonic lethality or midlarval arrest in *atfs-1(tm4525)* mutant animals (Figure S5A). We also performed the experiment using double RNAi (*afts-1(RNAi)+mma-1(RNAi)*) and did not observe any obvious synthetic lethality or arrest (Figure S5B). This indicates that in response to *mma-1(RNAi)*, mitochondrial hyperfusion is essential for viability while ATFS-1-dependent UPR^mt^ is not.

## DISCUSSION

Depending on the type and the severity of the stress to which mitochondria are exposed, different mito-chondrial stress responses (such as UPR^mt^, mitochondrial hyperfusion or mitophagy) can be activated in order to protect mitochondrial function [23]. Here, we show that reducing *LRPPRC* or *mma-1* function leads to an evolutionary conserved co-activation of UPR^mt^ and mitochondrial hyperfusion. Depletion of LRPPRC or MMA-1 protein results in a decrease in the production of mitochondria-encoded subunits of complex IV and thereby causes a reduction of complex IV activity [11, 14, 15]. Depletion of LRPPRC or MMA-1 protein also leads to an imbalance between nuclear-encoded and mitochondria-encoded subunits of this complex. We show that the activation of the UPR^mt^ occurs when this balance and, hence mitochondrial proteostasis, is disrupted (Figure 2; day 3 of *LRPPRC* siRNA). This finding suggests that the accumulation of unassembled complex IV subunits is most likely the signal that triggers UPR^mt^. Furthermore, our data indicate that UPR^mt^ is transiently activated until mitochondrial proteostasis is restored (Figure 2; day 5 of *LRPPRC* siRNA). We, therefore, propose that the transient activation of UPR^mt^ participates in the restoration of mitochondrial proteostasis by either proteolytic degradation of unassembled subunits by ClpP or assisted folding of nascent subunits by HSP70. In addition, the response may also involve the down-regulation of the genes encoding nuclear-encoded subunits of complex IV.

Whereas UPR^mt^ and mitochondrial hyperfusion in response to *LRPPRC mma-1* RNAi follow similar kinetics, genetic experiments in *C. elegans* indicate that the pathways that mediate these two responses are distinct. Specifically, *LRPPRC mma-1* RNAi-induced UPR^mt^ is dependent on the previously described HAF-1, ATFS-1 pathway, whereas *LRPPRC mma-1* RNAi-induced mitochondrial hyperfusion is not. This notion is furthermore supported by the fact that in *C. elegans*, a mild depletion of MMA-1 protein activates only the UPR^mt^ pathway whereas a strong depletion of MMA-1 activates both pathways. The pathway through which a reduction in *LRPPRC mma-1* function induces mitochondrial hyperfusion remains to be elucidated. Similarly, the mechanism through which mitochondrial hyperfusion and UPR^mt^ are coordinated is currently unknown.

**Figure 5:**
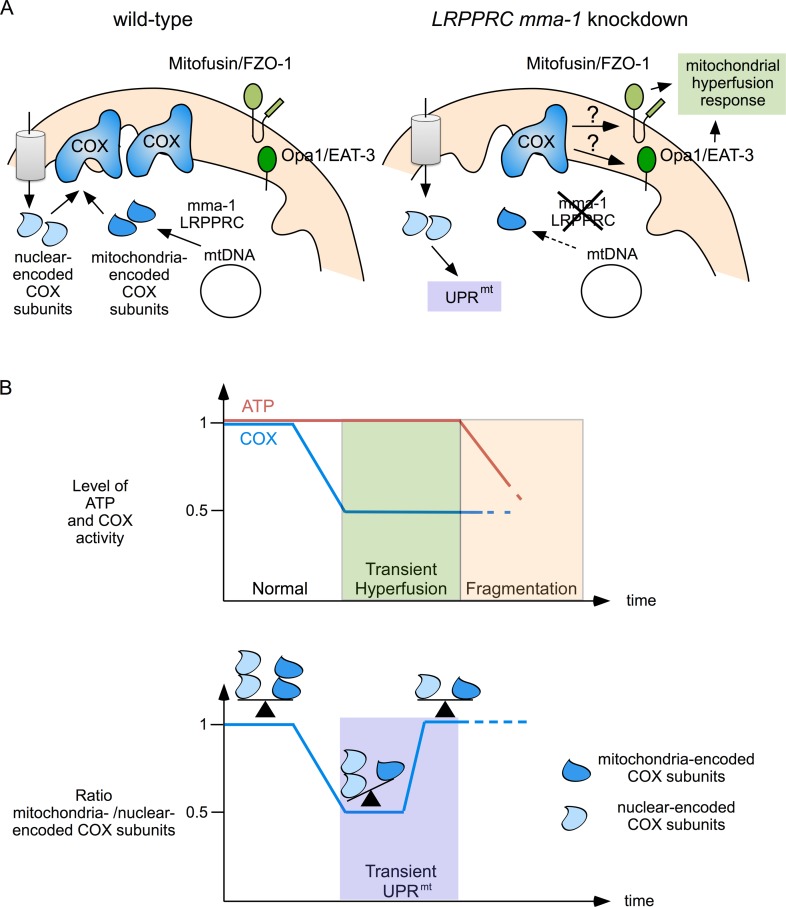
Mitochondrial hyperfusion and UPR^mt^ act together to protect mitochondrial function **(A)** Complex IV of the ETC is composed of mitochondria-encoded and nuclear-encoded subunits. Upon *LRPPRC mma-1* RNAi, the level of mitochondria-encoded subunits decreases, leading to a reduction in complex IV activity, which triggers the mitochondrial hyperfusion response and an accumulation of unassembled nuclear-encoded subunits, which triggers UPR^mt^. (**B**) While mitochondrial hyperfusion helps to maintain ATP level in response to a reduction in complex IV activity [11], UPR^mt^ helps to restore the balance between mitochondria-encoded and nuclear-encoded subunits of this complex. Mitochondrial hyperfusion can only compensate for reduced complex IV activity for a limited time. Prolonged inactivation of *LRPPRC mma-1* eventually leads to mitochondrial fragmentation and drop in ATP level as previously shown [11].

We propose that the mitochondrial hyperfusion response and UPR^mt^ act together to maintain and restore mitochondrial function (Figure 5). We have previously shown that mitochondrial hyperfusion helps to maintain cellular ATP levels despite a reduction in complex IV activity caused by *LRPPRC mma-1* RNAi [11]. We now propose that this transient compensation enables the cell to restore the balance between mitochondria-encoded and nuclear-encoded subunits of complex IV. However, as previously shown, prolonged inactivation of *LRPPRC mma-1* eventually leads to mitochondrial fragmentation, a drop in ATP level and the loss of cellular and organismal functions [11]. The two mitochondrial stress response pathways we unraveled are therefore likely to be important for the maintenance of mitochondrial function in response to moderate and short-term changes in mitochondrial proteostasis. Interestingly, UPR^mt^ as well as an ‘elongation’ of mitochondria are also induced in response to the infection of *C. elegans* by *Pseudomonas aeruginosa* [24]; however, how these two stress responses are induced in this context remains to be determined. In contrast, UPR^mt^ and mitochondrial hyperfusion may not protect against more drastic and long-term changes in mitochondrial proteostasis, such as in *C. elegans* in which *mma-1* has been chronically inactivated or in French Canadian Leigh Syndrome patients carrying mutations in the *LRPPRC* gene. In cell lines derived from these patients, mitochondria are fragmented [15], which indicates that the mitochondrial hyperfusion response has already failed. Whether UPR^mt^ is activated in these cell lines is currently unknown. In case it is activated, it will be interesting to determine whether this pathway contributes to the pathophysiology of this neurodegenerative disorder, which has not been completely elucidated yet.

We previously showed that the mitochondrial hyperfusion response is essential for viability in response to *mma-1(RNAi)* [11]. In contrast, we present evidence that ATFS-1-dependent UPR^mt^ is not essential for viability upon inactivation of *mma-1*. Furthermore, we show that ATFS-1-dependent UPR^mt^ is also not essential for viability in response to *spg-7(RNAi)*. One possible explanation for this finding is the existence of an ATFS-1-independent UPR^mt^ pathway. Indeed, Haynes and co-workers have shown that while *spg-7(RNAi)* induces the up-regulation of 685 genes, 294 of these genes are still up-regulated in *atfs-1(tm4525)* mutant animals [22]. Hence, in the context of *mma-1* inactivation, the ATFS-1-independent UPR^mt^ in combination with the mitochondrial hyperfusion response may be sufficient for the completion of embryonic and larval development.

The effect of the activation of UPR^mt^ on aging remains controversial. While Houtkooper *et al* reported an extension of lifespan in response to the activation of UPR^mt^ [7], Bennett *et al* observed no clear correlation between UPR^mt^ and lifespan [25]. We and others have previously shown in *C. elegans* and in rat that mitochondrial fragmentation increases with age and that this fragmentation is accompanied with a loss of mitochondrial volume [26, 27]. Interestingly, a recent study reports that fibroblasts isolated from long-lived human individuals exhibit hyperfused mitochondria [28]. While the genetic determinants resulting in this change in mitochondrial morphology remain unknown, the mitochondrial hyperfusion in the cells of these individuals has been shown to increase mitochondrial mass by preventing mitophagy [28]. Consequently, while the activity of their individual mitochondria decreases with age, the overall mitochondrial function of their cells is maintained, implicating a beneficial role of mitochondrial hyperfusion in aging [28]. In conclusion, a better understanding of UPR^mt^ and the mitochondrial hyperfusion response has not only the potential to increase our knowledge of how cells respond to mitochondrial stress under physiological and pathophysiogical conditions (i.e. French Canadian Leigh Syndrome) but also during aging.

## MATERIALS AND METHODS

### General *C. elegans* methods and strains

*C. elegans* strains were cultured as previously described [29]. Bristol N2 was used as the wild-type strain. Mutations used in this study were described by Riddle and co-workers [30] except: (LG IV) *haf-1*(*ok705*) (OMRF Knockout Group); (LG V) *atfs-1*(*tm4525*) (National BioResource Project). A summary of the transgenic lines is provided in Table S1.

### *C. elegans* RNA interference

RNAi by feeding was performed as previously described [11] with the following modifications. RNAi plates containing 1 mM IPTG were inoculated with 50 μl of 0.5 OD_600nm_/ml of concentrated *mma-1(RNAi)* or *spg-7(RNAi)* bacterial clones from the Ahringer library [31] or diluted 1:5 (v/v) with *mock(RNAi)* bacteria (HT115 bacteria transformed with the empty RNAi feeding vector pPD129.36) or *atfs-1(RNAi)* bacteria. 24 hours later, L4 larvae were inoculated on the RNAi plates and incubated at 20°C for 4 days. Mitochondrial morphology in L4 larvae of the F1 generation was analyzed by fluorescent microscopy as described [32]. For protein analyses, mixed-stage populations of worms were harvested and analyzed as described below. For the analysis of the effect of RNAi on *C. elegans* development, three wild-type (+/+), three *atfs-1(tm4525)* and 15 *fzo-1(tm1133)* L4 animals (15 animals of the *fzo-1(tm1133)* genotype were used since the broodsize of these animals is smaller) were inoculated on *mock(RNAi)*, *mma-1(RNAi) or spg-7(RNAi)* plates and incubated at 20°C for 18 hours. For the double RNAi experiment, three wild-type L4 larvae were inoculated on *mock(RNAi)*, *atfs-1(RNAi)*, *mma-1(RNAi)* (diluted 1:5 with *mock(RNAi)* or *atfs-1(RNAi)*) *or spg-7(RNAi)* (diluted 1:5 with *mock(RNAi)* or *atfs-1(RNAi)*) plates and incubated at 20°C for 18 hours. The adults were then transferred onto freshly seeded RNAi plates and incubated for an additional three hours at 20°C. After removing the adults, the embryos laid during these three hours were counted. 24 hours later, the number of unhatched embryos was counted to determine the embryonic lethality. The number of animals that reached adulthood by day 5 and the number of animals that underwent a midlarval arrest were also counted.

### Mammalian cell culture and transfection

SH-SY5Y cells were cultivated in DMEM:F12 supplemented with 15% FBS (Sigma), 1% non essential amino acids and 1% penicillin/streptomycin (Invitrogen); HEK293T cells were cultivated in DMEM supplemented with 10% FBS (Sigma) and 1% penicillin/streptomycin (Invitrogen); both cell lines were passaged twice a week and kept at 37°C with 5% CO_2_. For RNA interference, cells were transfected reversely with control stealth siRNA medium GC or stealth siRNA oligos targeting *LRPPRC* using Lipofectamine RNAiMax in OPTI-MEM (Invitrogen). 24 hours after transfection, fresh culture medium (25 mM glucose) was added. For the analysis of mitochondrial morphology, cells were kept in normal culture medium until harvested. For the analysis of UPR^mt^, the normal culture medium was replaced with medium containing only 5 mM glucose 24 hours before analysis.

### Protein analysis

Rabbit polyclonal anti–HSP-6 antibodies were generated using the antigenic peptide C^472^QEAKTAEEPKKEQN (cysteine plus HSP-6 C-terminal sequence) by Thermo Fisher Scientific, as previously described [33] and used at 1:5000 for Western analysis. Worm protein extracts were subjected to SDS/PAGE and Western. To detect HSP-60, GFP and α-Tubulin, we used a mouse monoclonal antibody developed by Nonet and co-workers and available at DHSB [20] (1:2000), rabbit polyclonal antibodies from Abcam ab290 (1:2000) and a mouse monoclonal antibody from Abcam ab7291 (1:2000), respectively. Images were quantified using the ChemiDoc XRS+ System (Bio-Rad). The data presented are ratios relative to the *mock(RNAi)*. Only samples for which *mma-1* knock-down was at least 20% were used for the statistically analysis. Statistical analysis was performed by using one sample *t*-test. Normality of the data was assessed by Shapiro-Wilk Normality test.

SH-SY5Y and HEK293T cells were harvested with pre-warmed 1x Laemmli-sample buffer and boiled for 10 minutes at 95°C and then subjected to SDS/PAGE and Western. The following antibodies were used: mouse monoclonal anti-β-Actin (Sigma AC-15, 1:5000), mouse monoclonal anti-LRP130 (sc-166178, 1:1000), goat polyclonal anti-HSP60 (sc-1052, 1:2000), rabbit polyclonal anti mortalin/mitochondrial HSP70 (sc-13967, 1:1000), rabbit polyclonal anti-ClpP (sc-134496, 1:1000), goat polyclonal anti-COXI (sc-48143, 1:500) and goat polyclonal anti-COXIV (sc-69359, 1:1000) (With the exception of the anti-β-Actin, all the antibodies were provided from Santa Cruz Biotechnology).

### Analysis of mitochondrial morphology in SH-SY5Y cells

Mitochondrial morphology was analyzed as previously described with the following modifications [11]. Cells grown on glass coverslips were fixed with PBS 3.7% PFA at 20°C for 10 minutes and permeabilized with PBS 0.2% Triton X-100 for 10 minutes at 20°C. The coverslips were blocked with PBS 5% BSA for 1 hour at 4°C and incubated overnight at 4°C in PBS 5% BSA with polyclonal anti-TOM20 antibodies (1:2000, Santa Cruz Biotechnology). After three washes with PBS, the coverslips were incubated with an Alexa555 goat anti-rabbit antibody (1:2000, Molecular Probes) in PBS 5% BSA for 2 hours at 20°C. After three washes with PBS, three washes with PBS 0.2% Tween and three washes with PBS, the glass coverslips were mounted on glass slides with MOWIOL 4–88 containing DAPI (Sigma). At least 300 cells per coverslip were counted in a blinded manner. Results are based on three independent experiments.

## SUPPLEMENTARY INFORMATION FIGURES AND TABLE


