# Supplementary material for: The loss of LRPPRC function induces the mitochondrial unfolded protein response
Source: Aging (Albany NY). 2015 Sep 26;7(9):701–12. doi: 10.18632/aging.100812 (PMC4600627; doi:10.18632/aging.100812)
Supplement: Supplementary file 1 [file aging-07-701-s001.pdf]

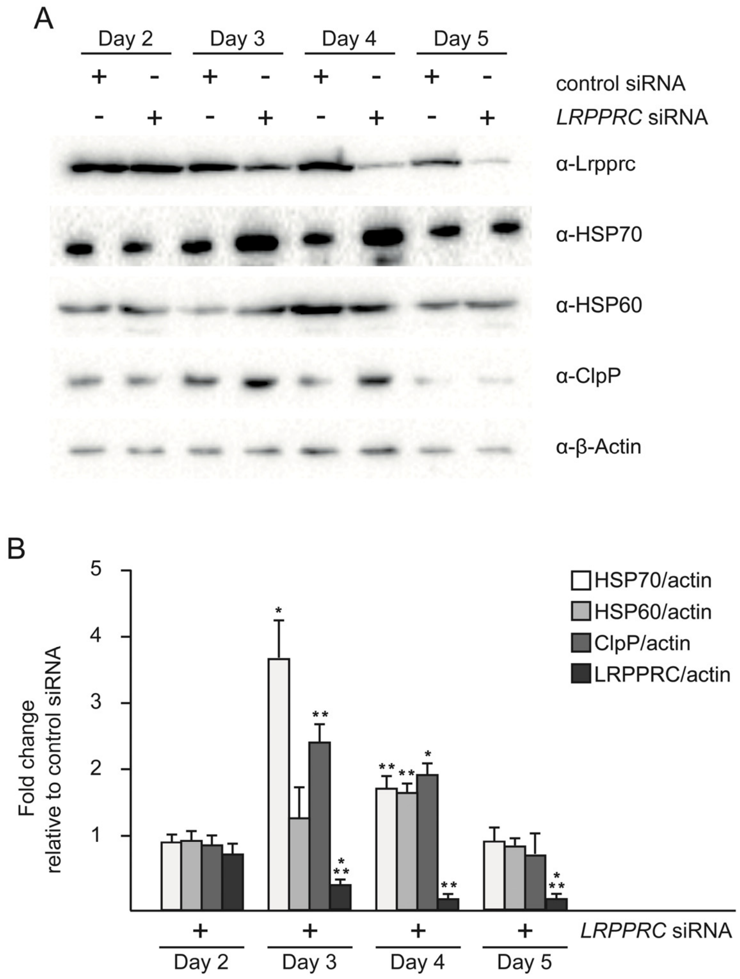

Figure S1. Silencing of *LRPPRC* also induces UPR^{mt} in HEK293T cells. UPR^{mt} in HEK293T cells treated with control or *LRPPRC* siRNA for 2, 3, 4 or 5 days and transferred for 24 hours into low glucose medium. Total protein extracts were then analyzed by Western using anti-LRPPRC, anti-HSP70, anti-HSP60, anti-ClpP and anti-β-Actin antibodies. Quantifications were performed on data from three independent experiments (For all panels, average values are shown and error bars indicate s.d.; * p≤0.05, ** p≤0.01 and *** p≤0.001 by one sample t-test).

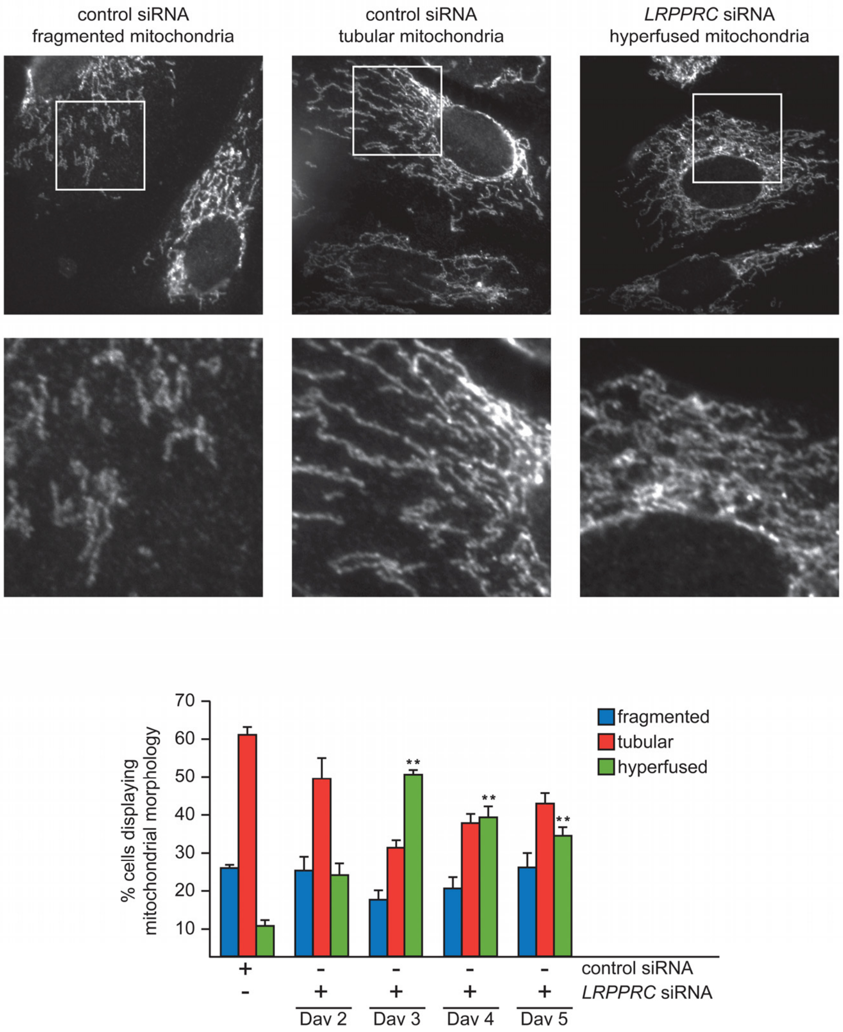

Figure S2. Silencing of *LRPPRC* induces mitochondrial hyperfusion in SH-SY5Y cells. Mitochondrial morphology of SH-SY5Y cells treated with control or *LRPPRC* siRNA for 2, 3, 4 or 5 days. Representative mitochondrial morphologies visualized using an anti-Tom20 antibody are indicated. Quantifications are based on data from three independent experiments. (n=300 cells per condition and experiment; average values are shown and error bars indicate s.d.; * p≤0.05, ** p≤0.01 and *** p≤0.001 by one way ANOVA).

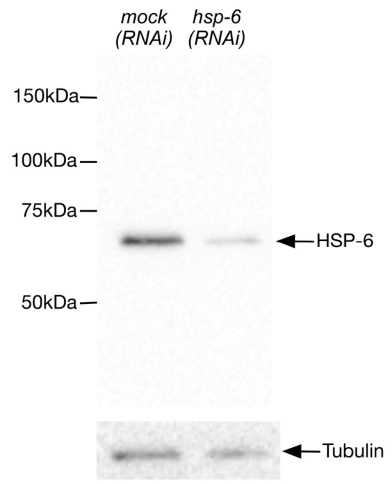

Figure S3. Rabbit polyclonal anti-HSP-6 antibodies recognize specifically *C. elegans* HSP-6 protein. L4 larvae of N2 (wild-type) were inoculated onto *mock(RNAi)* or *hsp-6(RNAi)* plates. 4 days later, animals of the F1 generation were lysed in Laemmli buffer and analyzed by Western using anti-HSP-6 and anti-Tubulin antibodies.

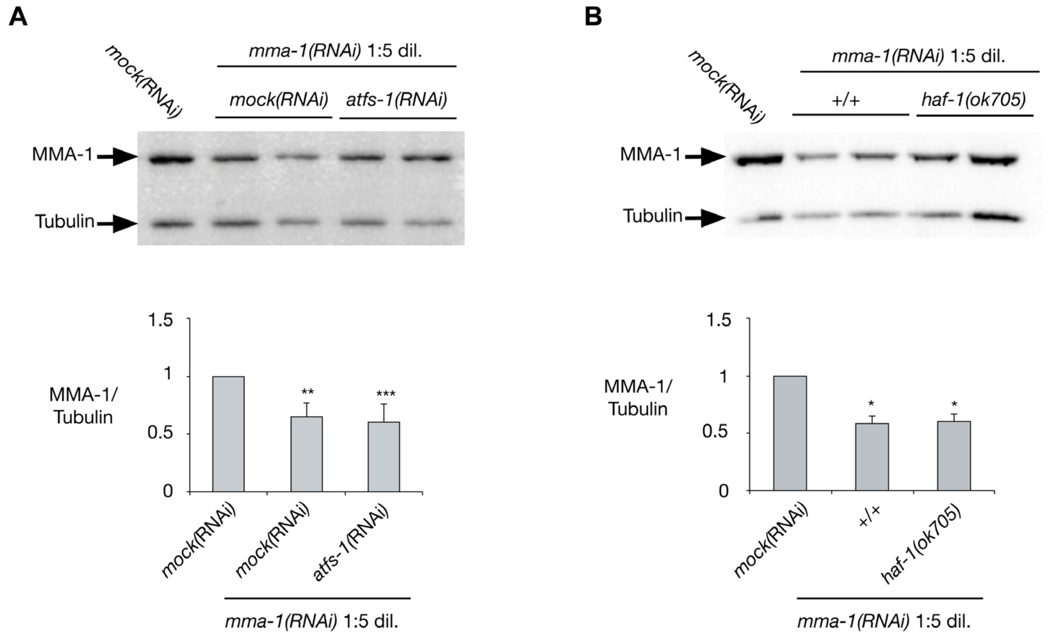

Figure S4. *atfs-1(RNAi)* and *haf-1(ok705)* do not affect *mma-1(RNAi)* efficiency. (A) L4 larvae of SJ4100 (*zcIs13 [$P_{hsp\text{-}6}$GFP]*) were inoculated onto *mock(RNAi)*, *mma-1(RNAi) or atfs-1+mma-1(RNAi)* plates. 4 days later, animals of the F1 generation were lysed in Laemmli buffer and analyzed by Western using anti-MMA-1 and anti-Tubulin antibodies. Ratios of MMA-1/Tubulin relative to the *mock(RNAi)* treated animals are indicated (n=5). **(B)** L4 larvae of SJ4100 (*zcIs13 [$P_{hsp\text{-}6}$GFP]*) or MD3550 (*haf-1(ok705); zcIs13[$P_{hsp\text{-}6}$GFP]*) were inoculated onto *mock(RNAi)* or *mma-1(RNAi)* plates. 4 days later, animals of the F1 generation were lysed in Laemmli buffer and analyzed by Western using anti-MMA-1 and anti-Tubulin antibodies. Ratios of MMA-1/Tubulin relative to the *mock(RNAi)* treated animals are indicated. (n=5). (For all panels, average values are shown and error bars indicate s.d.; * $p \leq 0.05$, ** $p \leq 0.01$ and *** $p \leq 0.001$ by one sample t-test).

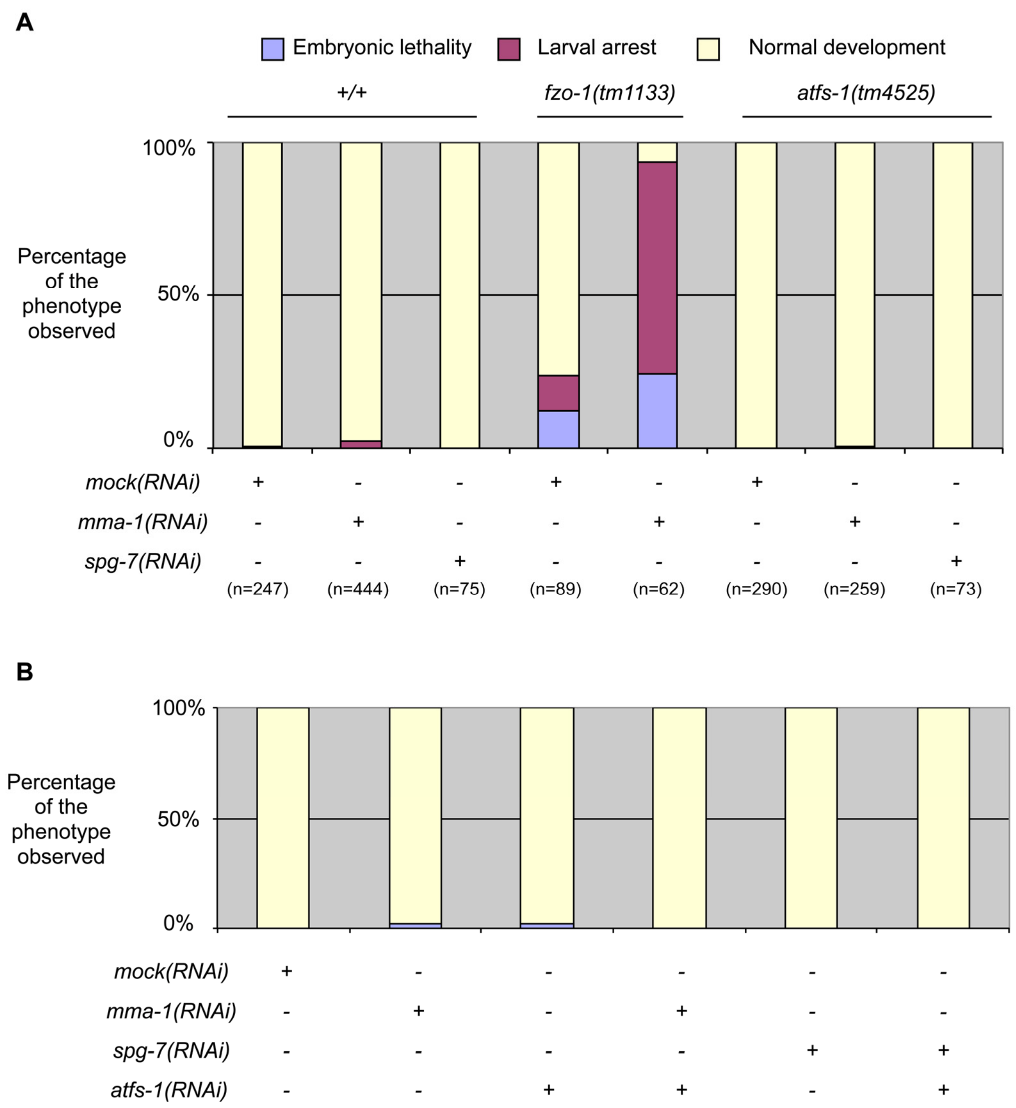

Figure S5. ATFS-1-dependent UPR^{mt} is not essential for viability in response to *mma-1*(*RNAi*). **(A)** Wild-type (+/+), *fzo-1(tm1133)* or *afts-1(tm4525)* L4 larvae were inoculated on *mock(RNAi)*, *mma-1(RNAi)* or *spg-7(RNAi)* plates. After 18 hours, a three hours lay off was performed onto new RNAi plates. The percentage of embryonic lethality, larval arrest as well as the percentage of animals developing into adults (normal development) was quantified in all conditions. **(B)** Wild-type L4 larvae were inoculated on *mock(RNAi)*, *atfs-1(RNAi)*, *mma-1(RNAi)* (diluted 1:5 with *mock(RNAi)* or *atfs-1(RNAi)*) or *spg-7(RNAi)* (diluted 1:5 with *mock(RNAi)* or *atfs-1(RNAi)*) plates. After 18 hours, a three hours lay off was performed onto new RNAi plates. The percentage of embryonic lethality, larval arrest as well as the percentage of animals developing into adults (normal development) was quantified in all conditions.

Table S1: List of the *C. elegans* transgenic lines used in this study.

Strain	Genotype	Reference
SJ4100	*zcIs13 V [$P_{hsp\text{-}6}$GFP]*	[18]
SJ4058	*zcIs9 V [$P_{hsp\text{-}60}$GFP]*	[18]
SJ4005	*zcIs4 V [$P_{hsp\text{-}4}$GFP]*	[18]
MD3550	*haf-1(ok705) IV ; zcIs13 V [$P_{hsp\text{-}6}$GFP]*	This study
MD3011	*bcIs78 I [$P_{myo\text{-}3}$mitoGFP]*	[11]
MD3572	*bcIs78 I [$P_{myo\text{-}3}$mitoGFP]; haf-1(ok705) IV*	This study
MD3573	*bcIs78 I [$P_{myo\text{-}3}$mitoGFP];atfs-1(tm4525) V*	This study
